# Novel Triamcinolone Acetonide-Loaded Liposomal Topical Formulation Improves Contrast Sensitivity Outcome After Femtosecond Laser-Assisted Cataract Surgery

**DOI:** 10.1089/jop.2019.0032

**Published:** 2019-11-04

**Authors:** Alejandro Gonzalez-De la Rosa, Jose Navarro-Partida, Juan Carlos Altamirano-Vallejo, Gerardo Daniel Jauregui-Garcia, Ricardo Acosta-Gonzalez, Miguel Angel Ibanez-Hernandez, Guadalupe Fernando Mora-Gonzalez, Juan Armendáriz-Borunda, Arturo Santos

**Affiliations:** ^1^ Tecnológico de Monterrey, Escuela de Medicina y Ciencias de la Salud, Zapopan, Mexico.; ^2^ Centro de Retina Médica y Quirúrgica, S.C., Centro Médico Puerta de Hierro, Zapopan, Mexico.; ^3^ Centro Médico Puerta de Hierro, Zapopan, Mexico.; ^4^ Instituto de Biología Molecular y Terapia Génica, Centro Universitario de Ciencias de la Salud, Universidad de Guadalajara, Guadalajara, Mexico.

**Keywords:** femtosecond laser-assisted cataract surgery, liposomes, visual outcomes, contrast sensitivity, macular edema

## Abstract

***Purpose:*** To assess visual results, macular modifications, and the incidence of clinically significant macular edema (CSME) in patients using a topical triamcinolone acetonide-loaded liposomal formulation (TA-LF) after femtosecond laser-assisted cataract surgery (FLACS).

***Methods:*** Fifty-six eyes after FLACS were selected. Twenty-eight eyes in the combined therapy group (P + N) were treated with prednisolone 1% and nepafenac 0.1% for 21 days postoperatively, whereas 28 eyes in the TA-LF group received a liposomal formulation containing 2 mg/mL of TA (0.2%) for the same period of time. Follow-up visits at 1 day, 6 weeks, and 12 weeks after surgery consisted of visual acuity, contrast sensitivity (CS), central foveal thickness (CFT), total macular volume (TMV) measurements, and the detection of CSME.

***Results:*** CS improved in the TA-LF group (basal value: 1.087 ± 0.339 vs. 1.276 ± 0.147 at week 12, *P* = 0.0346), whereas in the P + N group, CS was not different from the baseline (basal value: 1.130 ± 0.331 vs. 1.274 ± 0.133 at week 12, *P* = 0.1276). There were similar increases in postoperative CFT and TMV in both groups. CFT and TMV significantly correlate with CS only in the TA-LF group. The *r^2^* for CFT and CS was 0.1963 (*P* = 0.0206), whereas the *r^2^* for TMV and CS was 0.3615 (*P* = 0.0007) at 12 weeks. No difference was observed in the incidence of CSME between the groups.

***Conclusion:*** TA-LF is associated with better CS outcomes compared to combined therapy after FLACS.

## Introduction

State of the art cataract surgery allows improvement in the results and safety of lens surgery. An example of technological advancement in surgical lens techniques is femtosecond laser-assisted cataract surgery (FLACS). A femtosecond laser is an infrared laser (wavelength of 1053) that works by photodisruption. In recent years, the femtosecond laser has been used to perform the vital steps of cataract surgery: corneal incision, anterior capsulotomy, and lens fragmentation.^1,2^

FLACS technique has grown in popularity because it has refractive and visual outcomes comparable to those of conventional phacoemulsification cataract surgery, but with less phacoemulsification energy, anterior chamber inflammation, and corneal endothelial cell loss.^3^ However, these potential advantages of FLACS are not necessarily in accordance with patient requests.

Presently, patients demand excellent visual results after lens surgery that may significantly impact their quality of life,^4^ but limited examinations of visual outcomes are routinely made.^4,5^ Visual acuity (VA) is the most frequently utilized test for evaluating the overall performance of the visual system in the clinic setting.^4,5^ To obtain a more comprehensive evaluation of vision and its impact on the quality of life, physicians currently need to add separate tests such as those of contrast sensitivity (CS), glare testing, backlit acuity testing, random light scattering, and visual function questionnaires.^5^ Among all visual performance tests, that of CS has been found to be notably predictive of visual performance, but limited analysis of this visual outcome has been made in the context of FLACS.^6–8^

In contrast, despite the technological advancement in cataract surgery, pseudophakic cystoid macular edema (PCME) remains the most common cause of decreased central VA following an uneventful procedure. The incidence of clinical PCME (defined by symptomatic vision loss and evidence of cystoid macular edema) is reported between 0.1% and 3.8%^9–14^; however, the incidence of PCME can be as high as 10.9% when diagnosed by optical coherence tomography (OCT).^15^

Although previous studies on FLACS versus phacoemulsification cataract surgery have demonstrated less increase in central macular thickness and reduced anterior chamber flare^16,17^ with FLACS,^18^ clinical PCME can still happen. The reported prevalence of clinical PCME (also called clinically significant macular edema or CSME) related to FLACS is about 0.8%^19^ and it could be comparable to the published rates of clinical PCME in conventional phacoemulsification cataract surgery.

The ideal treatment to prevent PCME has not been established. Corticosteroids and topical nonsteroidal anti-inflammatory drugs (NSAIDs), either as monotherapy or combined, have proven to be useful^20–23^ and are broadly used as first-line drugs.^24^ A recent study, including 914 nondiabetic patients who underwent conventional phacoemulsification cataract surgery, demonstrated that the combination of topical bromfenac 0.09% and dexamethasone 0.1% was related to a lower incidence of CSME at 12 weeks postoperatively (1.5%) compared to monotherapy (bromfenac 0.09%, incidence of 3.6%; dexamethasone 0.1%, incidence of 5.1%).^25^ This comprehensive study considered VA as its main visual outcome; however, the quality of vision was not analyzed.

We consider that, in accordance with the scope of modern cataract surgery and visual results demanded by patients, not only is it important to achieve excellent VA outcomes after cataract surgery but also great visual quality. The prevention of CSME and exceptional CS results are matters to be solved by topical anti-inflammatory therapies as well. Recently, an innovative topical triamcinolone acetonide-loaded liposomal formulation (TA-LF) was used to efficiently deliver triamcinolone (TA) into the vitreous and the retina of rabbits^26^ and its therapeutic efficiency was verified in patients with refractory PCME.^27^ We performed this clinical assay to evaluate visual outcomes, particularly those related to CS, macular modifications, and the incidence of CSME, using TA-LF as an anti-inflammatory therapy in patients that underwent FLACS.

## Methods

### Study design

A randomized controlled trial was conducted to assess visual outcomes, macular changes, and the incidence of CSME, applying a TA-LF or the combination of topical prednisolone 0.1% and nepafenac 0.1% after FLACS; this study was conducted at a private-based retina specialty center in Guadalajara, Mexico (Centro de Retina Médica y Quirúrgica, S.C.). The Institutional Review Board (IRB)/Ethics Committee approval was obtained before the enrollment of patients (COFEPRIS 173300410A0035/2017). The study procedures were performed in accordance with the tenets of the Declaration of Helsinki. Written informed consent was obtained from all patients after full explanation of the nature and potential consequences of the study.

### Patients

Patients 45 years old or older who required FLACS and posterior chamber intraocular lens implantation in at least one eye were enrolled 7–14 days before surgery. Demographic and baseline clinical examinations, including central foveal thickness (CFT) and total macular volume (TMV) measurements by OCT, were gathered for enrolled patients, 1–3 days before surgery. Only patients who underwent uncomplicated cataract surgeries were included.

Exclusion criteria comprised the use of topical steroids or topical NSAIDs 1 month before the beginning of the study, placement of ocular steroid implants 12 months before study enrollment, use of intraocular corticosteroids or antiangiogenic drugs 3 months before the study, ocular diseases preventing adequate examination of the ocular fundus, any ocular disease that could be responsible for decreased VA (such as diabetic retinopathy, vascular occlusion, and macular degeneration), ocular hypertension, glaucoma, and unstable systemic disease, including systemic hypertension, diabetes mellitus, and any previous eye disease resulting in a medical history of macular edema. Patients with a previous cerebrovascular accident or myocardial infarction were also excluded.

### Surgical techniques and assigned therapy

All patients had the same preoperative and intraoperative treatment for both eyes. Surgeries were performed under topical anesthesia with tetracaine hydrochloride 5% (Ponti Ofteno, Sophia Labs., Zapopan, Mexico). Tropicamide 0.8% with phenylephrine hydrochloride 5% (T-P Ofteno, Sophia Labs.) was used to maintain dilation of the pupil during the entire surgery. Patients underwent FLACS, receiving the laser portion of the procedure first.

The LenSx laser system (Alcon, Inc.) was applied to perform anterior capsulotomy and lens fragmentation, and, subsequently, 3 clear corneal incisions were made: one temporal main incision and 2 paracentesis side ports (one of them was made to accommodate a second instrument, while the other one was used to inject viscoelastic into the eye in a more secure way). A suction ring was applied first.

Central round capsulotomy was created with energy set at 9 μJ and a diameter of 5.0 mm. A combined circular (2 circles, diameter of 2.0 mm and 2.8 mm) and 4-cut cross-shaped pattern (diameter of 8.0 mm) was used for lens fragmentation with a pulse energy of 10 μJ. The width of the main port corneal incision was 2.2 mm and 1.0 mm for the side port. The main port was located at 210° for the right eye and 30° for the left eye. Side ports were placed at 95° and 275° for the right eye and 100° and 280° for the left eye.

Following femtosecond laser treatment, patients were transferred to the operating room for phacoemulsification. After sedation, corneal incisions were opened, and the detached anterior capsule was removed with forceps. All other following steps were similar to those of conventional phacoemulsification. Patients received the Alcon AcrySof^®^ IQ PanOptix™ Presbyopia-Correcting Intraocular Lens.

After surgery, patients were randomly assigned to one of 2 treatment groups. Patients in the P + N group received prednisolone 1% 4 times per day and nepafenac 0.1% 3 times per day for 21 days postoperatively, whereas patients in the TA-LF groups received a liposomal formulation containing 2 mg/mL of TA (0.2%) 4 times per day for 21 days postoperatively. No other ocular corticosteroid or NSAID was allowed during the course of the study. All patients received postoperative gatifloxacin 0.3% 4 times per day for 14 days (Zymar, Allergan, Irvine, CA).

### Visual outcome assessment

Visual outcomes of TA-LF 0.2% compared to P + N combined therapy were evaluated with VA and CS. The best corrected visual acuity (BCVA) was measured using an ETDRS chart at 4 m and expressed as the logarithm of the minimum angle of resolution (logMar). CS was evaluated by the Pelli-Robson CS test. The obtained values of logarithmic CS (1/contrast), as well as VA values, were recorded at each study visit. Study visits were scheduled at 1 day, 6 weeks, and 12 weeks after surgery.

### Macular changes and cystoid macular edema assessment

To analyze macular changes, CFT and TMV were calculated by OCT (Cirrus OCT Carl Zeiss, Meditec, Dublin, CA) at each study visit. The occurrence of PCME and CSME was investigated at weeks 6 and 12. PCME was defined as a mean increase in central subfield macular thickness of 10% or more over baseline with cystic changes on the OCT. Cystoid changes and other retinal pathologies were identified by 2 independent, masked retina specialists. CSME was defined as PCME with less than a 0.2 logMAR BCVA improvement compared with the preoperative baseline.^25^

### Safety and tolerability assessment

Tolerability was assessed through the collection and summary of ocular and nonocular adverse events (AEs), serious AEs, ocular assessments, and vital signs, whether volunteered by the enrolled patients or discovered by study site personnel during questioning or by any other means. In addition, ocular surface staining with fluorescein, intraocular pressure (IOP), slit lamp, and anterior and posterior segment evaluations was executed at each visit to identify ocular AEs.

Subjects were withdrawn from the study if they presented any evidence of poor tolerability or any AE, for example, corneal ulcers, corneal opacities, epithelial defects, anterior chamber inflammation (cell/flare), and conjunctival and/or episcleral injection related to the use of this topical formulation. AEs were assigned standard codes/terms for the event based upon the MedDRA Coding dictionary version 18.1.

### Rescue treatment

Rescue treatment was contemplated when patients developed CSME during the course of the study. Patients with CSME were excluded from the study and continued with topical therapy for CSME. If CSME persisted after 8 weeks of topical treatment, patients received one intravitreal injection of 4 mg of preservative-free TA. IOP lowering drugs were considered when the recorded IOP was ≥22 mmHg or >4 mmHg compared to that of the contralateral eye.

### Preparation of liposomal formulation

OPKO Health, Inc. (Guadalajara, Jalisco, Mexico) provided the TA-LF. Preparation of TA-LF was carried out as formerly described.^26^ Concisely, self-forming, thermodynamically stable TA loaded liposomes (QuSomes^®^) were spontaneously generated upon adding polyethylene glycol glyceryl dimyristate (PEG-12) to an aqueous solution containing TA. The composition of TA-LF is described in Table 1. The final TA concentration in the resulting dispersion was 2 mg/mL (0.2%).

**Table 1.** Triamcinolone Acetonide Loaded Liposomal Formulation Composition

*Reagent*	*Volume*
Triamcinolone acetonide	2.0 mg
Kolliphor HS 15	50 mg
PEG-12 glyceryl dimyristate	100 mg
Ethyl alcohol	14 μL
Citric acid anhydrous	0.8 mg
Sodium citrate dihydrate	4.675 mg
Benzalkonium chloride	0.1 mg
Grade 2 purified water	Q.S.1.0 mL

### Statistical analysis

Quantitative variables were described using mean and standard deviation. Qualitative variables were described using frequencies and percentages. Mann–Whitney U and Wilcoxon signed-rank tests were performed for the analysis of age, BCVA, CS, CFT, TMV, and IOP on dependent and independent samples, respectively. For the analysis of the gender and study eye, a Fisher exact test was performed. Significance was defined as a *P* value under 0.05. All data were analyzed using SPSS 22.0 software (IBM SPSS Statistics for Macintosh, Version 22.0. Armonk, NY: IBM Corp.).

## Results

Fifty-six eyes were included. Twenty-eight eyes were assigned to each group. Regarding age, gender, or the treated eye, no statistically significant differences were observed between the groups. The clinical characteristics of patients for the P + N combined therapy and the TA-LF groups are summarized in Table 2. Twenty-seven eyes from the P + N group completed the 12-week follow-up since one patient required rescue treatment for CSME at week 6. All patients in the TA-LF group completed the follow-up period.

**Table 2.** Clinical Characteristics of the Groups

	*P + N*	*TA-LF*	
	n = *28 eyes*	n = *28 eyes*	P
Age	59.39 ± 4.3	58.39 ± 5.7	0.5488
Gender			
F	16 (0.57)	18 (0.64)	0.5845
M	12 (0.43)	10 (0.36)	
Eye			
OS	14 (0.5)	13 (0.46)	0.7884
OD	14 (0.5)	15 (0.54)	

F, female; OS, left eye; M, male; OD, right eye; P + N, Prednisolone 1% + Nepafenac 0.1% group; TA-LF, triamcinolone acetonide-loaded liposomal formulation containing 2 mg/mL of TA group.

**FIG. 1.**
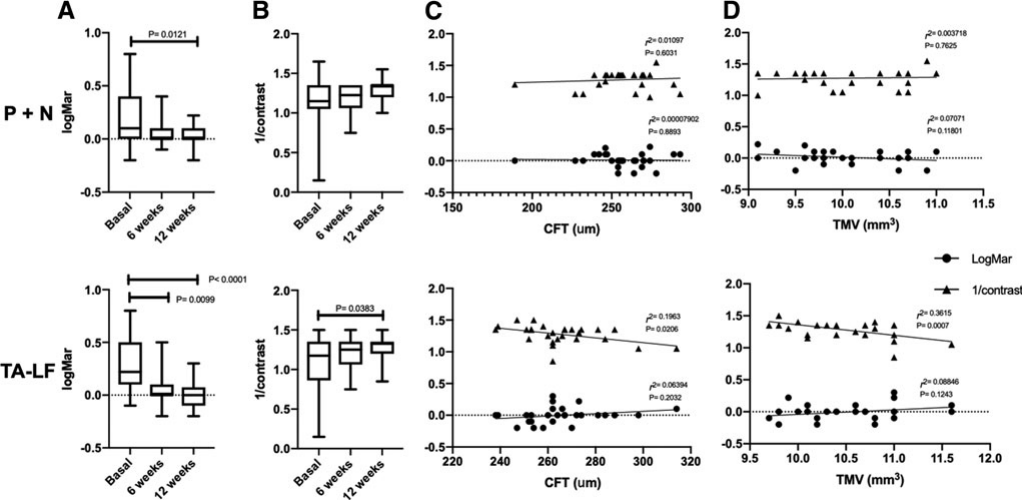
Visual outcomes of combined (P + N) and TA-LF therapies. Visual acuity **(A)**, contrast sensitivity **(B)**, and correlations of CFT **(C)** and TMV **(D)** with visual outcomes are presented. Visual acuity improves significantly in both groups **(A)**; however, contrast sensitivity improvement achieved statistical significance only in TA-LF group **(B)**. CFT and TMV correlates with CS just in the TA-LF group at 12 weeks (inferior rows, **C **and** D**). CS, contrast sensitivity; CFT, central foveal thickness; P + N, Prednisolone 1% + Nepafenac 0.1% group; TA-LF, group exposed to triamcinolone acetonide liposomal formulation; TMV, total macular volume.

Concerning the analysis of visual outcomes within the groups, CS improved over time in the TA-LF group (basal value: 1.087 ± 0.339 vs. week 12: 1.266 ± 0.147, *P* = 0.0346), while in the P + N group, CS was not different from baseline (basal value: 1.130 ± 0.331 vs. week 12: 1.274 ± 0.133, *P* = 0.1276) (Table 3 and Fig. 1A, B). Remarkably, patients treated with TA-LF had the BCVA and CS compared to the P + N group; however, these findings were nonstatistically different in the analysis between groups (Table 4).

**Table 3.** Differences Within Groups in Visual Acuity, Contrast Sensitivity, Macular Thickness, and Total Macular Volume

	*Basal*	*6 weeks*	*12 weeks*	P *value*
Visual acuity (logMar)				
P + N	0.176 ± 0.235	0.058 ± 0.118	▲0.011 ± 0.106	0.0143
TA-LF	0.252 ± 0.248	▲0.03 ± 0.142	▲0.005 ± 0.136	<0.0001
Contrast sensitivity (1/contrast)				
P + N	1.130 ± 0.331	1.291 ± 0.160	1.274 ± 0.133	0.1276
TA-LF	1.087 ± 0.339	1.217 ± 0.191	▲1.266 ± 0.147	0.0346
CFT (μm)				
P + N	249.42 ± 25.78	261.42 ± 30.87	256.55 ± 20.79	0.3574
TA-LF	256.21 ± 15.16	▲266.42 ± 16.06	▲265.92 ± 18.55	<0.0001
TMV (mm^3^)				
P + N	9.90 ± 0.65	10.00 ± 0.79	10.04 ± 0.54	0.6386
TA-LF	10.08 ± 0.71	▲10.55 ± 0.52	▲10.50 ± 0.52	<0.0001
IOP (mmHg)				
P + N	16.07 ± 2.60	▲13.67 ± 2.38	▲14.17 ± 1.76	0.001
TA-LF	15.71 ± 2.20	▲12.89 ± 2.69	▲13.50 ± 1.93	0.0193

CFT, central foveal thickness; IOP, intraocular pressure; logMar, logarithm of the minimal angle of resolution; TMV, total macular volume; ▲, *P* < 0.05 comparing with basal values.

**Table 4.** Differences Between Groups in Visual Acuity, Contrast Sensitivity, Macular Thickness, and Total Macular Volume

*Parameter*	*P + N*	*TA-LF*	P *value*
Visual acuity (logMar)			
Baseline	0.176 ± 0.235	0.252 ± 0.248	0.2467
6 weeks	0.058 ± 0.118	0.030 ± 0.142	0.4194
12 weeks	0.011 ± 0.106	−0.005 ± 0.136	0.8370
Contrast sensitivity (1/contrast)			
Baseline	1.130 ± 0.331	1.087 ± 0.339	0.6343
6 weeks	1.291 ± 0.160	1.217 ± 0.191	0.7347
12 weeks	1.274 ± 0.133	1.276 ± 0.142	0.8344
CFT (μm)			
Baseline	249.42 ± 25.780	256.214 ± 15.164	0.2351
6 weeks	261.42 ± 30.874	266.428 ± 16.065	0.4504
12 weeks	256.55 ± 20.794	264.857 ± 17.080	0.0832
TMV (mm^3^)			
Baseline	9.903 ± 0.657	10.085 ± 0.711	0.3242
6 weeks	10.007 ± 0.797	10.557 ± 0.52	0.0034
12 weeks	10.044 ± 0.546	10.503 ± 0.52	0.0023
IOP (mmHg)			
Baseline	16.07 ± 2.60	15.71 ± 2.20	0.5775
6 weeks	13.67 ± 2.38	12.89 ± 2.69	0.2512
12 weeks	14.17 ± 1.76	13.40 ± 1.90	0.1798

As expected, there was an increase in postoperative CFT and TMV in both groups, but only the TA-LF group reached significance (Table 3). Nevertheless, these parameters were nonstatistically different in the analysis between groups (Table 4).

TA-LF was well tolerated throughout the entire study period. Neither ocular (increased intraocular pressure and ocular surface abnormalities) nor systemic AEs were documented. None of the eyes exhibited increments in IOP, and no IOP lowering drugs were required. In fact, a significant reduction in IOP was observed in both groups. IOP levels decreased from 16.07 ± 2.60 to 13.67 ± 2.38 at week 6 in the P + N group, while they decreased from 15.71 ± 2.20 to 12.89 ± 2.69 at week 6 in the TA-LF group (Table 3). There were no signs of irritation nor surface complications related to the study formulation until the end of the follow-up period in any of the subjects.

Outstandingly, CFT and TMV correlate significantly with CS only in the TA-LF group. The *r^2^* for CFT and CS was 0.1675 (*P* = 0.0306), whereas the *r^2^* for TMV and CS was 0.2605 (*P* = 0.0055) at week 6. This outcome remained until the end of the follow-up period. The *r^2^* for CFT and CS was 0.1963 (*P* = 0.0206), while the *r^2^* for TMV and CS was 0.3615 (*P* = 0.0007) at week 12. The correlation between macular modifications and visual outcomes is presented in Table 5 and graphically depicted in Fig. 1C and D.

**Table 5.** Correlation Between CFT and TMV with Visual Acuity and Contrast Sensitivity in P + N and TA-LF Groups

	*6 weeks*				*12 weeks*			
	*Visual acuity (logMar)*		*Contrast sensitivity (1/contrast)*		*Visual acuity (logMar)*		*Contrast sensitivity (1/contrast)*	
	r^2^	P	r^2^	P	r^2^	P	r^2^	P
P + N								
CFT	0.3294	0.0014	0.05844	0.2152	0.0007902	0.8893	0.01097	0.6031
TMV	0.005192	0.7156	0.1355	0.0539	0.07071	0.1801	0.003718	0.7625
TA-LF								
CFT	0.1036	0.0948	0.1675	0.0306	0.06394	0.2032	0.1963	0.0206
TMV	0.06301	0.1976	0.2605	0.0055	0.08846	0.1243	0.3615	0.0007

Finally, we did not find any difference in the incidence of PCME and CSME between groups. The incidence of PCME in the P + N and the TA-LF groups at week 6 was 10.7% and 3.5%, respectively; in contrast, the incidence of CSME was 3.5% and 0%, respectively (Table 6). The odds of developing CSME were not significantly higher in the P + N group than in the TA-LF group (Table 6). One patient with CSME in the P + N group required rescue treatment.

**Table 6.** Incidence of CME and CSME and Odds Ratio Values

	*P + N*	*TA-LF*		
*Parameter*	*Incidence n/N (%)*	*Incidence n/N (%)*	*OR (95% CI)*^a^	P
CME within 6 weeks	3/28 (10.7)	1/28 (3.5)	3.24 (0.316–33.22)	0.6040
CME within 12 weeks	2/27 (7.4)	1/28 (3.5)	2.16 (0.184–25.31)	0.9747
CSME within 6 weeks	1/28 (3.5)	0/28 (0)	3.10 (0.121–79.64)	0.4930
CSME within 12 weeks	0/27 (0)	0/28 (0)	1.03 (0.019–54.08)	0.9859

Odds of developing CME and CSME in the P + N group are presented.

CI, confidence interval; CME, cystoid macular edema; CSME, clinically significant macular edema; OR, odds ratio.

## Discussion

Although there are different factors that determine the visual performance (eg, CS, temporal processing speed, and dark/light adaptation), VA is the most frequently utilized test to evaluate the function of the visual system. However, CS testing has been found to be notably predictive of visual performance in the natural world. VA testing quantifies the ability of the visual system to resolve fine details at a specific distance using high contrast letters of varying sizes, whereas CS refers to the ability of the visual system to discriminate edges in a scene and effectively define the boundaries of objects.^5^ The CS function has the potential of adding more information about the functioning of the visual system than that provided by VA because it assesses sensitivity over a wide range of spatial frequencies, while VA primarily measures sensitivity at high spatial frequencies.

Despite the value of the CS test, it is not usually evaluated. In the context of FLACS, the CS outcome has poorly been explored.^6–8^

In a recent study accomplished in patients who underwent presbyopic refractive lens exchange surgery, a minimal but significant difference in CS for the spatial frequency of 18 cycles/° was found between the FLACS and the conventional phacoemulsification lens extraction (CLE) groups, with the best outcome in the CLE group. In addition, statistically significant differences in uncorrected near visual acuity (UNVA) between groups were found at 12 months postoperatively. Although the difference was small in magnitude, UNVA was significantly worse in the FLACS group compared to the CLE group at the end of the follow-up^6^ period. Differences observed between the groups in the study are discreet; however, they become of great relevance in the framework of modern cataract surgery, where exceptional visual results are the target.

As we have already exposed, visual outcomes, particularly those related to CS, improved over time with the use of TA-loaded liposomes. CS basal value in the TA-LF group was 1.087 ± 0.339 versus 1.276 ± 0.147 at week 12 (*P* = 0.0346), while in the P + N group, CS at week 12 was not different from baseline (basal value: 1.130 ± 0.331 vs. 1.274 ± 0.133 at week 12, *P* = 0.1276). Moreover, we found that only in the TA-LF group, CFT and TMV significantly correlate with the visual outcome for CS. These results support the idea that TA-LF could be superior compared to combined therapy regarding CS outcomes, an issue of great interest in the context of modern cataract surgery, where exceptional visual results are demanded by patients.

We hypothesized that the improvement in CS in the TA-LF group over the P + N group had to do with the ability of liposomes to efficiently deliver steroid into the retinal tissue.^26,28^ It has been established that the adequate function of photoreceptors is crucial for CS^29,30^ and it is known that macular edema causes the dysfunction of foveal photoreceptor cells (reduced directional sensitivity of photoreceptor cells).^31^ In contrast, steroids could improve the function of photoreceptors and protect them from damage through the activation of the protein kinase B (AKT) signaling pathway.^32–34^ Therefore, steroids could presumably improve the photoreceptor function in patients with PCME and, consequently, improve the results of the CS test.

The crucial event that supports our hypothesis is the arrival of bioactive concentrations of steroids to the retina. Different reports have proved that liposomes are suitable vehicles for the delivery of steroids into the retina when topically applied.^26,28^ Besides, the pharmacokinetic study of TA-LF validated that this formulation was capable of efficiently releasing TA into the vitreous and the retina when topically applied in rabbits.^26^

In addition, it is important to emphasize that components of TA-LF formulations other than liposomes can increase the efficiency of the delivery of TA into the vitreous and the retina. For example, polyethylene glycol (15)-hydroxystearate or Kolliphor^®^ HS 15 is a constituent of TA-LF; this reagent is a potent nonionic solubilizer and emulsifying agent with low toxicity proposed to act as a permeability enhancer that would promote drug transport across cell membranes (increasing the endocytosis rate) and improve drug translocation throughout the paracellular route (it affects actin organization in the cell cytoskeleton with subsequent tight junction opening).^35^

Unfortunately, only a few reports have explored the relationship between CS outcomes and the use of anti-inflammatory ophthalmic formulations in patients with PCME. For example, Ginsburg et al., reported in 1995 that patients who underwent manual extracapsular extraction improved their CS scores using ophthalmic formulations of flurbiprofen 0.03% or indomethacin 1%. In contrast, Heier et al. (2000) described that the combined therapy of ketorolac tromethamine 0.5% and prednisolone acetate 1% was related to improved VA and CS, as well as to the leakage on fluorescein angiography in patients with acute macular edema after phacoemulsification and posterior chamber intraocular lens implantation.^36,37^

To the best of our knowledge, our study is the first report to significantly correlate the use of anti-inflammatory compound with CS outcomes after FLACS. However, confirmatory studies with bigger sample sizes are required.

In addition to CS, we reviewed the activity of TA-LF on macular changes and the incidence of PCME and CSME. Briefly, we did not find any difference in the frequency of PCME and CSME between the TA-LF and combined therapy groups. Although one case of CSME occurred in the P + N group (compared with the TA-LF group with an incidence of 0%), no statistically significant differences were observed. We could assume that TA-LF is at least as effective as a combined therapy in preventing CSME associated with FLACS. Nevertheless, compared to previous studies using other topical steroids, TA-LF remains therapeutically superior. In any case, the incidence of PCME is lower with TA-LF.

The reported incidence of clinical PCME with the use of conventional topical steroids is 4–5.1%,^22,25^ whereas the incidence of CSME with TA-LF is 0%. We demonstrated that topical TA-LF prevents the appearance of macular edema as efficiently as the combination of topical NSAIDs and steroids. This enhanced activity of topical steroids in TA-LF is presumably attributed to the liposomal content of the formula, which allows ocular barriers to be overcome.

Liposomes (LPs) are particles composed of an aqueous core, delimited by a membrane-like lipid bilayer, that work as carriers for water-soluble, lipid-soluble, and amphiphilic drugs.^38–41^ LPs are nontoxic, low antigenic, easily metabolized, and biodegradable^42^ and have been used to improve drug transport and bioavailability in ocular tissues.^43,44^

Recently, in animal models, Jin Li et al. (2019) validated that eye drops containing TA-loaded liposomes are an efficient method to deliver this drug into the posterior segment of the eye,^28^ which supports previous findings published by our group.^26^ Even though Li et al. reached higher TA entrapment efficiency in their TA-loaded liposomes prepared through the calcium acetate gradient method, in our experience, this characteristic does not compromise the therapeutic activity of TA-LF.

Previous studies have established that the topical combination of steroids and NSAIDs is superior in preventing CSME^25^ to any drug alone. For instance, in a retrospective study on the prevention of PCME (defined as new or worsening of anatomic macular edema or its thickening demonstrated by OCT), its postoperative rate in patients receiving prednisolone acetate 1% and dexamethasone sodium phosphate 0.1% was 4.0% and 4.1%, respectively.^22^ However, in a large prospective study, the combination of topical bromfenac 0.09% and dexamethasone 0.1% was linked to a lower incidence of CSME at 12 weeks postoperatively (1.5%) compared to bromfenac 0.09% (incidence of 3.6%) and dexamethasone 0.1% (incidence of 5.1%).^25^

The stronger therapeutic effect of the combined therapy could be explained by the synergetic mechanism of action of these drugs. The therapeutic activity of corticosteroids consists of 3 elements: the blockage of leukotriene and the synthesis of prostaglandins (PGs) through the inhibition of phospholipase A2 in the arachidonic acid cascade, the reduction of macrophage and neutrophil migration, and the decrease in capillary permeability and vasodilation. In contrast, NSAIDs inhibit cyclooxygenase enzymes (COX-1 and COX-2). Both enzymes catalyze the biosynthesis of eicosanoids from arachidonic acid to produce PGs and thromboxanes, triggering vasodilatation and the disruption of the blood-ocular barrier.^24^

Poor therapeutic activity of conventional topical steroids in PCME could be explained by the limited concentration of the drug reaching the posterior ocular segment due to both ocular and blood-retinal barriers. Intravitreal injections of steroids reach proper intraocular concentrations and avoid ocular barriers.^45^ In fact, intravitreal TA has proven to be suitable for refractory PCME, as well as for the treatment of other vitreoretinal diseases.^46–52^ Different reports using intravitreal triamcinolone acetonide (IVTA) have shown high efficacy with significant improvement of VA and a substantial reduction in macular thickness in patients with refractory PCME.^49–52^ Unfortunately, IVTA is related to increased IOP, which requires topical lowering drugs in most cases,^49,52^ and to other severe complications such as endophthalmitis, lens injury, and retinal detachment.^53–55^

In addition to intravitreal injection, other routes for steroid administration have been used to treat PCME. For example, systemic steroid treatment proved to be effective for refractory PCME and was associated with prompt response and resolution^56^; however, due to a high rate of systemic side effects, it is not considered as an accepted therapeutic strategy. Periocular steroids (retrobulbar and sub-Tenon injections) are efficient in treating refractory PCME and have been associated with improved VA, but intraocular pressure rise remains a common side effect.^57^ In a recent study, patients with refractory PCME were treated with sub-Tenon triamcinolone injections, which elicited an improvement in BCVA and CFT; however, this therapeutic strategy was less effective compared to that of topical nepafenac.^58^

Finally, intravitreal biocompatible implants containing dexamethasone (Ozurdex ©), which were designed to slowly release 0.7 mg over up to 6 months, have been successfully used to treat refractory PCME.^59–62^ However, studies with the dexamethasone implant have stated that intraocular pressure rise still occurs, but this circumstance is less frequent and more moderate than after triamcinolone injections. TA-LF has also been used to treat refractory PCME with excellent results. TA-LF topical therapy was related to an improvement in BCVA and a reduction in CFT in patients with refractory PCME, but without inducing an increase in IOP.^27^

In fact, an important observation made in our trial was that IOP rises were not documented in any of the study groups. Conversely, IOP significantly decreased after cataract surgery. This phenomenon was not unexpected because it is well known that cataract surgery reduces the ocular pressure from 1.5 to 4 mmHg.^63^ The mechanism is not clear, but it seems to be related to anatomical and physiological changes induced by lens surgery.^64^ Besides, small doses of TA topically used (100 μg of TA per drop) might probably prevent the well documented IOP rise induced by steroids.

In synthesis, TA-LF has proved to deliver TA into the vitreous cavity and the retina, evading ocular barriers.^26^ Moreover, it has been demonstrated that this formulation is efficient in the management of refractory PCME,^27^ and, as our results have already shown, it is also useful in the prophylactic treatment of PCME without inducing a rise in IOP or the appearance of side effects.

We consider that potential concerns that may arise from our data revolve around the observed incidence of CSME and the presumable systemic therapeutic effect of TA through the topical instillation of TA-LF. It is important to mention that the incidence of CSME observed in our study (3.5%) for combined therapy (P + N group) differs from that reported by other authors; such is the case of the incidence of 1.5% reported by Wielders et al. (2018),^25^ who used bromfenac 0.09% and dexamethasone 0.1%. Comparing the incidence of clinical PCME after prophylactic medical therapy in different reports has been difficult to date. This difficulty is mainly caused by the variations in the evaluated patient populations, study designs, surgical techniques, topical drugs used, therapy periods, and, most importantly, in the methods used to evaluate macular thickening.^65^

To achieve more valid comparisons between studies, we used the diagnosis criteria of the European multicenter trial on the prevention of cystoid macular edema after cataract surgery in nondiabetics (ESCRS PREMED), one of the most extensive studies on medical prophylaxis for CSME that included 914 patients from 12 study centers.^25^ Either way, as we previously mentioned, the incidence of CSME in the TA-LF group was 0%, which in any case is inferior to that reported for preventive monotherapy or combined therapy in the different articles, including the ESCR PREMED study, and it suggests the exceptional efficacy of TA-LF. However, further studies with larger sample sizes and longer follow-up periods should be considered to ratify this finding.

Regarding the potential systemic therapeutic effect through the topical instillation of TA-LF, it is important to consider the following evidence. With most ophthalmic formulations, only a small fraction of an eye drop dose will reach the posterior segment after topical administration.^66–68^

In general, < 5% of topically applied doses enter the eye through transcorneal permeation or permeation through the conjunctiva and the sclera. However, most of the applied dose (50%–95%) is systemically absorbed through the conjunctiva of the eye or through the nose (after the drug enters the lacrimal drainage system).

The posterior segment of the eye may receive drugs from topically applied eye drops, either through topical absorption into the eye or by systemic absorption through the bloodstream.^69,70^ However, it is important to notice that the experiments on the contribution of systemic drug return to the ocular tissues have been conducted in animal models, mainly in rabbits or small species. Therefore, the effect of drug reentry would probably be lower in humans because the volume of drug distribution is much greater in 70 kg humans than in 2 kg rabbits. Furthermore, the rabbit eye is smaller than the human eye and, therefore, topically absorbed drugs may reach the posterior segment more easily in rabbits than in humans.^71^

In any case, the reentry phenomenon was not explored in our patients, but it is unlikely that this will contribute to the therapeutic activity of TA-LF since one drop only contains 100 μg of TA for a large apparent volume of distribution. Moreover, the pharmacokinetic study on TA-LF validated that this formulation was capable of efficiently releasing TA into the vitreous and the retina when topically applied in rabbits.^26^

Finally, multiple efforts have been made to generate a topical formulation for the delivery of TA into the posterior segment of the eye using different delivery strategies such as those that include liposomes and lipid nanoparticles^26,28,72,73^; however, only TA-LF has been proved in clinical assays, where its efficiency and safety profiles have satisfactorily been demonstrated.^27^ Once again, in this study, we observed that TA-LF is well tolerated and holds an acceptable safety profile, with no ocular AEs.

In conclusion, TA-LF is at least as effective as a combined therapy for the prevention of CSME associated with FLACS. Nevertheless, it seems that its therapeutic activity could be superior, since TA-LF was related to better visual outcomes, specifically in terms of CS. CS has been proposed as a more reliable method to determine visual performance than VA^4,5^; therefore, the impact of TA-LF on the visual quality observed in this study could be of relevance for modern lens surgery techniques like FLACS. However, due to the sample size included in our report, additional studies should be considered.
